# Dissecting the regulatory network of transcription factors in T cell phenotype/functioning during GVHD and GVT

**DOI:** 10.3389/fimmu.2023.1194984

**Published:** 2023-06-27

**Authors:** Rebecca Harris, Mobin Karimi

**Affiliations:** Department of Microbiology and Immunology, State University of New York (SUNY) Upstate Medical University, Syracuse, NY, United States

**Keywords:** GVHD, GVL, allotransplantation, transcription factors, regulatory networks

## Abstract

Transcription factors play a major role in regulation and orchestration of immune responses. The immunological context of the response can alter the regulatory networks required for proper functioning. While these networks have been well-studied in canonical immune contexts like infection, the transcription factor landscape during alloactivation remains unclear. This review addresses how transcription factors contribute to the functioning of mature alloactivated T cells. This review will also examine how these factors form a regulatory network to control alloresponses, with a focus specifically on those factors expressed by and controlling activity of T cells of the various subsets involved in graft-versus-host disease (GVHD) and graft-versus-tumor (GVT) responses.

## 1 Introduction

Transcriptional control of immune gene expression and immune system functioning has been widely studied to better understand how these processes can shape immune responses to pathogens. However, it has become clear that in different contexts, the same transcription factors (TFs) can have vastly different (and often contradictory or unexpected) effects. For example, a factor which suppresses gene expression in one cell type may activate expression of the same genes in a different cell type. Beyond this, TFs can regulate each other in a complex network of activation, suppression, and enhancement, and disease states can alter their function. A significant amount of research is needed to not only understand the biology of these TFs and what genes are changing in a specific context, but also how those changes are regulated, and how the regulators are regulated in a network. As such, many elements of the immune system and its responses are only understood to a superficial level, and recent work has focused on deepening this knowledge.

**Figure 1:**
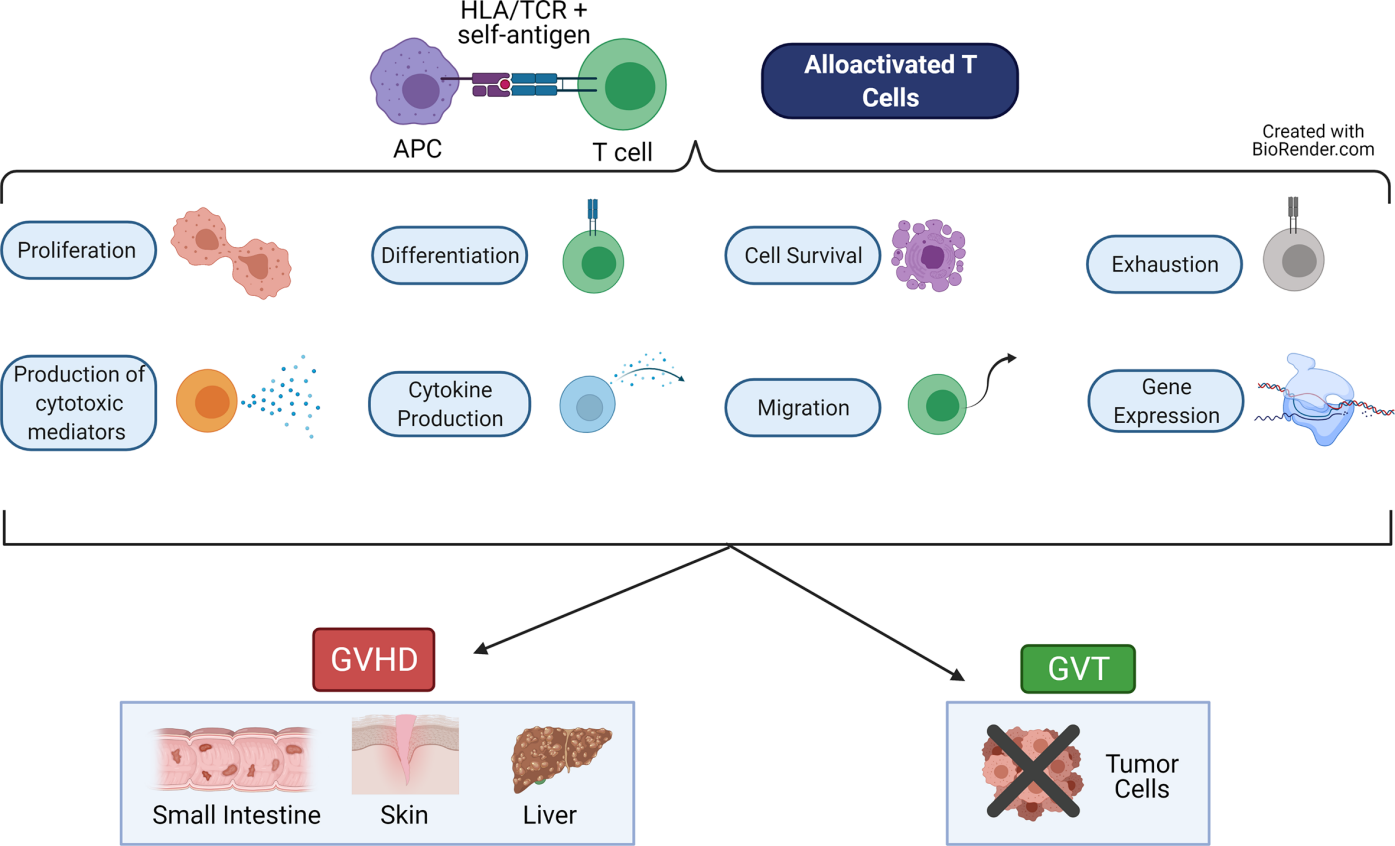
Major Functions of Alloactivated T cells. When alloreactive T cells recognize alloantigen (recipient self antigen) presented by an antigen presenting cell (APC), the T cell becomes alloactivated. These T cells then perform a variety of functions, including: proliferate, differentiate into cytotoxic cells, produce cytokines and cytotoxic mediators, migrate, become exhausted, survive or undergo apoptosis, and express altered gene programs. These T cell functions lead to graft-versus-host disease (GVHD), which damages healthy host tissues in the target organs (skin, small intestine, liver). These T cell functions also lead to the graft-versus-tumor (GVT, also called graft-versus-leukemia, GVL) effect, which eliminates residual malignant cells. Figure created using BioRender.com.

Graft-versus-host-disease (GVHD) and graft-versus-tumor (GVT) effects (also called graft-versus-leukemia or GVL effects) are intricately linked alloreactive processes driven primarily by naive T cells (1–3). These T cells are alloactivated by presentation of non-self-antigen by non-self HLA (MHC in mouse) molecules on the APCs of the host (4, 5). This results in proliferation of the T cells, differentiation to cytotoxic effector cells, migration to target organs or tumor sites, and release of cytokines and cytotoxic mediators (6, 7) (**Figure 1**). Each of these major functions are controlled by networks of transcription factors, which regulate expression of important genes used to perform these functions. While GVT effects help to rid the patient of residual malignant cells and prevent relapse of the cancer, GVHD damages the healthy host tissues (6, 8).

Thus, the main goal of research in this field is to eliminate or reduce GVHD while preserving or enhancing GVT effects. These two processes are performed using similar pathways in the same T cells, and both rely on the major functions described above (proliferation, migration, producing cytokines). Therefore, it is not possible to separate these effects simply by removing entire subsets of T cells without some sort of trade-off (such as increased infection rates post-transplant) (6, 8–12). Instead, current research focuses on manipulating the underlying regulatory networks or signaling pathways of the T cell, to drive the cell to perform one process but not the other. A deeper understanding of these regulatory networks is therefore critical to achieving these goals.

The use of various knockout, knockdown, or knock-in genetic models have led to a better understanding of how different transcription factors participate in alloactivated T cell functioning. Transcription factors are notoriously difficult to target using chemical/pharmaceutical means, although recent breakthroughs have broadened the possibilities in this area (13–15). Due to these difficulties, GVHD/GVT models typically rely on genetic manipulation to alter expression or functioning of the transcription factor. The sections below will summarize what has been discovered about a variety of critical factors in these processes.

This review seeks to summarize how TFs contribute to the functioning of mature alloactivated T cells, through study of graft-versus-host disease (GVHD) and graft-versus-tumor (GVT) effects. This review will also examine how these factors form a regulatory network to control alloresponses, with a focus specifically on those factors expressed by and controlling activity of T cells of the various subsets involved in GVHD/GVT.

## 2 Search strategy

Research articles and literature reviews were identified from the PubMed and Google Scholar databases using any of the following search terms:

“gvhd transcription factor”, “gvhd AND transcription factor”, “gvhd gvt transcription factor”, “gvhd gvl transcription factor”, “gvt transcription factor”, “gvl transcription factor”, “*name of factor* gvhd”, “*name* gvt”, “*name* gvl”, “*name* AND gvhd”, “*name* AND gvt”, “*name* AND gvl”, “alloactivation transcription factor”, “*name* alloactivation”

Articles were excluded if the article language was not English and no translation was available, if the full-text article was unavailable/irretrievable, if the factors under study were not considered transcription factors, and if the factor discussed was not expressed in T cells or the subject of study was not T cells (given that the focus of this review is on T cell-expressed factors). From the results of the search terms above, articles found to be relevant were included.

## 3 Transcription factors which impact but likely do not separate GVHD/GVL

### 3.1 Eomesodermin (Eomes)

Eomes is a transcription factor typically associated with T cell memory, and thought to balance T-bet, which is associated with effector T cells. Many research reports examine both Eomes and T-bet together, rather than separately, because they are seen to be intricately linked. Some reports suggested that both Eomes and T-bet are important for antitumor immunity, but these studies were done after adoptive transfer, not allogeneic transplant (16). However, the importance of Eomes in both GVHD and GVT effects was shown by another group, which used a mouse model of allogeneic hematopoietic stem cell transplantation (allo-HSCT) (17).

Lack of Eomes in T cells led to reduced GVHD severity and improved survival. Interestingly, loss of Eomes reduced GVHD more than loss of T-bet (will be discussed further below). This reduction in GVHD was primarily due to reduced CD8^+^ T cell migration to target organs, an increase in Tregs, and decreased cytokine production in CD4^+^ T cells. Proliferation and activation of CD4^+^ T cells were also reduced when Eomes was lost. Introduction of tumor cells showed that loss of Eomes disrupted GVT effects, suggesting that Eomes is critical for this effect. Experiments with mixed WT or Eomes T cells showed that Eomes is important for CD8-driven GVT, but not for CD4-driven help during allo-HSCT. Therefore, although Eomes was important for both functions, Eomes-deficient CD4^+^ T cells could be used to separate GVHD from GVT effects (17).

Histone deacetylase 11 (Hdac11) controls T cell phenotype through epigenetic changes. Loss of Hdac11 led to increased expression of Eomes and T-bet in T cells, as well as increased proliferation of the T cells. Hdac11 KO T cells also showed increased production of cytokines and cytotoxic mediators. These changes resulted in exacerbated GVHD in mice receiving Hdac11 KO T cells, but GVT effects were also enhanced. Therefore, loss of Hdac11 enhanced GVHD and GVT due to increased expression of Eomes (18). Another group showed that T(R)1 cells (Type 1 Tregs) are the most frequent type of Tregs in allo-HSCT, and these cells require expression of Eomes to develop. These cells are also important for reducing GVHD, suggesting a role for Eomes in suppressing GVHD. Expression of Eomes in these cells requires expression of T-bet, further complicating matters (19). Therefore, depending on the cell expressing it, Eomes may be helpful or harmful in GVHD.

Eomes also plays a role in GVT, but the exact role remains to be elucidated. Some reports suggest that Eomes is important for CD8^+^ T cell function during antitumor responses, primarily by promoting exhaustion. This suggests that Eomes would inhibit GVT effects. However, while loss of both Eomes alleles stopped development of tumor-attacking CD8^+^ T cells, loss of only one allele limited exhaustion in the T cells, which promoted antitumor immunity (20). These experiments were done in canonical antitumor models, so they may not directly apply to GVT. More recent work showed that Itk KO mice, which have higher expression of Eomes (but not T-bet), have reduced GVHD severity but maintained GVT function (12, 21). Therefore, Eomes appears to be critical for both GVHD and GVT, due to its effects on T cell migration and cytokine production.

### 3.2 Forkhead box P3 (Foxp3) and regulatory T cells (Tregs)

Foxp3 is the lineage-defining transcription factor for Tregs. Foxp3 was initially associated with GVHD when a study found that patients with GVHD had less expression of *Foxp3* mRNA. This group also showed that stimulating CD4+ T cells *in vitro* with self antigen increased expression of FOXP3, which in turn reduced proliferation of CD8^+^ T cells (22). This work showed that Foxp3-expressing CD4^+^ T cells could possibly be used to treat GVHD. A short time later, another group found that higher expression of FOXP3 among donor CD4^+^ T cells indicated a lower risk of GVHD development in the recipient. They also confirmed that in GVHD patients, Tregs were reduced (23). Tregs were found at a higher frequency in skin biopsies from patients with low grade treatment-responsive GVHD than in patients with more severe or non-responsive GVHD (24).

At this point, research began on whether Tregs could be used as a therapy. However, natural Tregs (nTregs, also called thymus-derived Tregs [tTregs]) are found in low numbers in the body, so iTregs (induced *in vitro* or in the periphery [pTregs] as opposed to in the thymus) were differentiated to try to provide a source for these cells. One study found that iTregs induced *in vitro* were limited in their ability to ameliorate GVHD symptoms because Foxp3 expression was not stable. After Foxp3 was lost from these cells, they reverted to an inflammatory state, with production of cytokines and contribution to damage (25). However, when iTregs were induced with antigen specificity, they were able to effectively prevent GVHD in recipient mice expressing that antigen (26).

Increasing stability of Foxp3 expression by blockade or loss of IL-27 increased the efficiency of iTregs at blocking GVHD. This treatment allowed better reconstitution of nTregs and iTregs, and also increased their suppressive ability (27). Vitamin C was also shown to stabilize Foxp3 expression in CD8^+^ iTregs, which can suppress GVHD but still control tumors with the GVL effect (28). Mice with GVHD that were given azacytidine showed reduced GVHD, largely due to increased Treg frequencies and expression of Foxp3 (29, 30). Furthermore, one group showed that CD4^+^ CD25- T cells could be engineered to ectopically express Foxp3, leading to a phenotype similar to nTregs which suppressed GVHD but preserved GVL effects (31).

More recently, SNPs in the *FOXP3* gene locus were found to be associated with GVHD outcomes. These SNPs were present at higher frequencies in patients with GVHD than in patients without GVHD. One of these SNPs was predictive of risk for aGVHD, when analyzed on its own. Interestingly, these SNPs were also associated with a lower risk of infection post-HSCT (32). These findings highlight a critical problem with allo-HSCT, which is that the same effector T cells responsible for GVHD are also the cells which fight off pathogens. Therefore, having fewer Tregs allows GVHD to occur, but also allows better targeting of pathogens because Tregs are not inhibiting the effector cells. Inhibition of Sirtuin-1 (Sirt-1) also affected Tregs in GVHD, promoting the differentiation of iTregs. This led to reduced GVHD but maintained GVT effects. Sirt-1 loss in iTregs stopped them from becoming cytotoxic T cells and increased the stability of Foxp3. Sirt-1 inhibition with Ex-527 enhanced survival while reducing GVHD severity (33).

Although Tregs are traditionally defined as CD4^+^ FOXP3^+^ CD25^+^, recent work has shown that other types of Tregs do exist. One of these is CD8^+^ Tregs, which are known to have similar functions compared to CD4^+^ Tregs, but are otherwise understudied. CD8^+^ Tregs also have transcriptional profiles similar to CD4^+^ Tregs, and different from other CD8^+^ T cells. However, murine studies of GVHD showed that these CD8^+^ Tregs were not able to suppress GVHD as well as CD4^+^ Tregs. This was mainly due to an increase in expression of Bim and less expression of Mcl-1. These expression changes made the CD8^+^ Tregs more proapoptotic than CD4^+^ Tregs, making them more likely to die. However, when Bim-deficient CD8^+^ Tregs were transplanted, they had better survival and stronger GVHD suppression than normal CD8^+^ Tregs (34).

These findings suggested that CD8^+^ Tregs could be used as therapy if their tendency towards apoptosis was addressed. Another approach to improve these CD8^+^ Tregs was to stabilize Foxp3 expression by deletion of Jak2. This allowed the Tregs to reduce GVHD but also maintained GVT effects. The CD8^+^ Tregs were not converted to cytotoxic cells as easily, and they maintained high Foxp3 levels, making them more stable and capable of suppression (35).

Double-negative suppressor/regulatory T cells (TCRα/β^+^ CD4/CD8-, called “DN T cells”) were recently discovered as well, and these cells can suppress GVHD in mouse models. Allo-HSCT patients also show low levels of DN T cells in severe GVHD cases, and high levels of DN T cells in mild cases. One group found that these DN T cells inhibit CD4^+^ T cell mTOR signaling. DN Tregs also change the metabolic program of CD4^+^ T cells to stop them from causing as much damage (36). Therefore, canonical Tregs use Foxp3 to suppress GVHD, and DN Tregs appear to also be suppressive during alloresponses.

### 3.3 B cell lymphoma 6 (Bcl6)

Bcl6 is a transcription factor important in Tfh cells and germinal center B cells. Its role in GVHD was only recently investigated. Mice which lack Bcl6 in T cells or in B cells are unable to form germinal centers. To investigate the role of this factor in chronic GVHD, one group used mice lacking Bcl6 in B cells (Bcl6 flox x MB1cre) to induce cGVHD in recipient mice. Mice receiving splenocytes from these Bcl6 cKO mice still experienced severe cGVHD symptoms, with no difference from mice receiving WT cells. Next, the group examined the impact of Bcl6 in T cells using a T cell-specific knockout (Bcl6 flox x CD4cre). Importantly, mice receiving Bcl6 cKO T cells had reduced cGVHD symptoms and histopathology, as well as reduced mortality. This occurred because Bcl6 deficiency prevented CD4^+^ effector T cells from expanding/proliferating (37).

These findings were recently confirmed by use of a Bcl6 inhibitor, 79-6, to inhibit cGVHD in mice. Interestingly, transplanted mice given this inhibitor had reduced cGVHD severity, but damage to the liver and colon was still seen, and Tfh numbers were not significantly changed. These data suggested that the Bcl6 inhibitor primarily prevented B cell effects. However, this group found that Bcl6 in both T cells and B cells was important for cGVHD development (38–40). Therefore, Bcl6’s role in T cells in cGVHD is to support development of Tfh cells which are capable of producing germinal centers, which are needed to cause disease, as well as to induce expansion of CD4^+^ effector T cells. It is unknown whether the same is true during aGVHD.

### 3.4 Basic leucine zipper transcription factor, ATF-like (Batf)

Batf drives differentiation of Th17 cells (41), which are involved in GVHD-induced damage. Recent reports found that this factor also impacts GVHD through control of IL-7-expressing T cells. Batf was found to induce differentiation of effector memory T cells which expressed IL-7 and GM-CSF, and were able to infiltrate peripheral tissues such as the colon. Mice receiving Batf-deficient T cells had milder GVHD and better survival compared to those receiving WT T cells. These Batf-/- T cells produced and induced less IL-6 when alloactivated. The GM-CSF/IL-7-producing T cells driven by Batf were not Th17 cells, and had a distinct phenotype from other cell populations present. These cells were critical for GVHD damage in the intestines (42). These data suggest that although Th17 cells do cause damage, they may not be solely responsible for GVHD phenotypes, and other cell subsets play a role. Targeting of Batf may provide benefit aside from Batf’s role in Th17 development (43).

### 3.5 Mothers against decapentaplegic homolog 3 (Smad3)

Smad3 is a transcription factor which is activated by TGF-β signaling. Generally, higher levels of SMAD3 were linked to less severe cases of GVHD (44). One group showed that loss of Smad3 in donor cells caused severe GVHD more frequently in an allo-HSCT mouse model, despite matching of major MHC markers. The intestines were the most impacted by GVHD damage, due to increased migration of Smad3 KO CD4+ T cells. These CD4^+^ T cells expressed T-bet, indicating their ability to act as effectors by releasing Th1 cytokines. These data suggest that increasing levels of Smad3 would actually be beneficial for GVHD outcomes (45).

Smad3 may also play a role in controlling allograft rejection of allotransplanted mouse hearts. However, in this case, survival of the transplanted hearts was improved when Smad3 was lost in the donor mouse, due to proinflammatory cytokine reduction and reduced migration of T cells into the tissue. Immune responses were shifted from Th1-driven to Th2-driven responses, and Th17 and Treg responses were also decreased. Thus, these results suggested that removing Smad3 would be most beneficial in allotransplant (46). However, in both studies, Smad3 was shown to control T cell migration, fate decisions, and cytokine production.

### 3.6 c-Fos/c-Jun (Ap-1) and c-Myc

c-Fos/c-Jun (Ap-1) and c-Myc are proto-oncogenes that encode transcription factors of the same name, which are important for proliferation of cells, especially in response to stimuli such as growth factors. c-Fos and c-Jun proteins form a heterodimer to create the Ap-1 complex, while c-Myc acts by itself (47, 48) c-Fos, c-Jun, and c-Myc are important early on for immune reconstitution (49).

Curcumin, a plant-derived phytochemical from the turmeric root, may attenuate GVHD. Treatment of allo-transplanted mice with curcumin reduced GVHD severity and pathology, mainly through reduced T cell proliferation and cytokine production. Tregs were also increased in curcumin-treated recipient mice. The expression of c-Fos and c-Jun was reduced in target tissues such as skin and intestine when the mice were given curcumin, suggesting that Ap-1 plays a role in mediating these processes. Therefore, downregulation of Ap-1 (c-Fos/c-Jun) was beneficial by reducing migration and cytokine production of T cells, as well as enhancing Tregs (50). However, a later study showed that in human HSCT patients, c-Myc and c-Jun protein levels were reduced. c-Jun expression was higher in short-term HSCT survivors than long-term, and c-Fos expression was lower in non-surviving patients (49). Therefore, it is still unclear whether these factors are beneficial or harmful during GVHD. However, they appear to play a role in T cell migration, Treg fate, and cytokine production.

## 4 Transcription factors which may separate GVHD/GVL

### 4.1 T-box transcription factor TBX21 (T-bet)

T-bet is also a transcription factor critical for T cell function, and is typically associated with effector T cells. Reports showed that loss of T-bet (which controls development of Th1 cells) resulted in reduced GVHD severity. When both T-bet and Rorγt (which controls Th17 cells) were lost, recipient mice showed no GVHD, as well as reduced migration to target organs, reduced Th1/Th17 cytokine production, and reduced organ damage. However, these T-bet/Rorγt DKO T cells had maintained GVT functions, separating GVHD from GVT (51). Our data showed that loss of T-bet in T cells increased expression of Eomes (52), and since Eomes is critical for GVT effects (17, 21), (this helps to explain the maintenance of GVT effects in the absence of T-bet.

Th1 cells were shown to be important in acute GVHD (aGVHD) in a rat model, and Th1 cell development is driven by T-bet (53, 54). Interestingly, loss of T-bet in donor cells led to increased pulmonary GVHD in allotransplanted recipients. This was because when Th1 cells were reduced due to loss of T-bet, Th2 and Th17 cells expanded and produced cytokines, leading to damage (55). Therefore, loss of T-bet alone may not prevent GVHD. In addition, in a mouse model of allogeneic heart transplant, loss of T-bet in T cells led to more rapid rejection of the allogeneic heart than loss of Rorγt (or WT T cells). Loss of both T-bet and Rorγt in T cells also caused rapid allorejection (56). Thus, loss of T-bet may not be protective in all situations of allogeneic responses.

More recently, work was done on loss of T-bet alone, rather than in combination with other deficiencies. This work showed that loss of T-bet in T cells led to reduced GVHD severity. This was due to reduced damage to organs by CD4^+^ T cells because of reduced migration to target organs, and reduced cytokine production. Loss of T-bet specifically in Th17 cells also reduced GVHD. Finally, T-bet deficient T cells that were allotransplanted showed reduced GVT effects, although neutralizing IL-17 fixed this defect (57). Loss of T-bet in allotransplant recipients also led to reduced GVHD severity (58). These results suggest that T-bet is important for migration and cytokine production during GVHD and GVT. Loss of T-bet in donor T cells in a model of chronic GVHD and autoimmunity led to an increase in Foxp3+ Tregs and reduced clinical signs after allotransplantation (59). Dual TCR T cells, which are increased in chronic GVHD (cGVHD), express T-bet, suggesting a role for T-bet even in cGVHD alloresponses (60). Therefore, T-bet plays a role in GVHD and GVT through control of migration and cytokine production, as well as through interactions with Eomes.

### 4.2 Retinoic-acid-receptor-related orphan nuclear receptor gamma, isoform T (Roryt)

Rorγt is a major lineage factor driving T cells to the Th17 fate. Due to the major role of Th17 cells in GVHD, Rorγt has been a factor of interest in GVHD research. Initial work showed that Th17 cells cause severe GVHD with increased migration to target organs and proliferation of alloreactive cells. However, loss of Rorγt in donor mice did not affect the ability of CD4^+^ T cells to cause GVHD. The group concluded that Rorγt-driven Th17 cells are sufficient, but not required for GVHD to occur (61). Loss of Rorγt was also shown not to affect alloresponse-driven colitis (intestinal GVHD), despite the role of Th17 cells in the process (62). (However, loss of both Rorγt and T-bet did reduce GVHD severity. Interestingly, GVT effects were maintained despite loss of both factors (63). Loss of both Batf and Rorγt also reduced intestinal GVHD (62). Other work showed that loss of Rorγt relieved GVHD severity in an MHC-matched model of bone marrow transplant (BMT), but had no effect in an MHC-mismatched model (64).

In contrast, another group showed that treatment of donors cells with G-CSF before MHC-mismatched BMT resulted in reduced skin GVHD, with no impact on other organs. This was due to reduced expansion of Rorγt+ T cells because of increased expression of Socs3, an inhibitory factor (65). In mice given a mismatched BMT and then treated with mesenchymal stem cells (MSCs) expressing Icos, GVHD severity was reduced in part because of reduced Rorγt expression (among other factors) (66). One group suggested that accumulation of Rorγt+ Th17 cells in target organs (67, 68) is due to increased expression and activity of Stat3, which is critical for Rorγt expression and Th17 fate (68).

Expression of heme oxygenase-1 (HO-1) also correlated with reduced GVHD because it affects expression of RORγT and Th17 fate choices (51). Most recently, ILCs expressing RORγT were found to be protective during intestinal GVHD, and were depleted in GVHD patients (69). Thus, the role of Rorγt still remains to be fully elucidated during GVHD, but T cells expressing this factor (Th17 cells) contribute to GVHD severity through increased migration and expansion. GVT effects do not seem to be affected by loss of Rorγt, so this factor may not be critical for antitumor immunity.

### 4.3 Signal transducer and activator of transcription (Stats)

Stat family transcription factors are activated by Jak factors (see below) in the Jak/Stat pathway. Stats are primarily involved in immune responses, proliferation, differentiation, and apoptosis (70). These factors have many roles in diverse contexts, and have been heavily studied in GVHD, where Stats 1, 3, 4, 5, and 6 have been shown to be important.

Stat1 was first identified as important in a study of GVHD using SAHA, an Hdac inhibitor. This treatment reduced the rapid Stat1 phosphorylation which occurred when GVHD was initiated, both in the liver and in the spleen. SAHA also reduced Stat3 phosphorylation. Use of this inhibitor resulted in less GVHD-driven mortality by lowering cytokine production of T cells (71). These data suggest that Stat1 (and possibly Stat3) is important for T cell cytokine production. Another group used Stat1-deficient mice to examine this factor’s role in GVHD. Again, GVHD severity was reduced in the absence of Stat1, primarily due to reduced activation and proliferation of donor T cells. Ablation of Stat1 also increased proliferation of Tregs, and inhibited apoptosis in these cells. These Tregs were hypersuppressive, helping to prevent GVHD damage (72).

One study sought to identify how and when Stat1 and Stat3 are activated during GVHD. After induction of GVHD, T cells (CD4^+^ and CD8^+^) with either Stat1 or Stat3 phosphorylation expand, followed by activation of Stat1/Stat3-driven gene expression (73). These data suggest that Stat1 and Stat3 are critical to GVHD progression. While examining IL-22-driven GVHD pathology, another group found that GVHD patients had increased STAT1 expression in the GI tract (74). Gain-of-function STAT1 mutations in humans also lead to autoimmunity and other problems, which were successfully corrected with HSCT in one patient. Before treatment, this patient showed lowered IL-17 responses, increased IFN-γ production, and increased phosphorylation of STAT1 (75). Therefore, Stat1 is critical for regulation of T cell cytokine production, activation, expansion, and Treg fate decisions.

Stat3 was first examined in GVHD using a flow cytometry technique that was novel at the time, using antibodies to detect phosphorylation within cells. This study showed that Stat3 phosphorylation occurred during GVHD(76), and these data were later confirmed (68, 77). Interruption of Stat3 phosphorylation with a small molecule inhibitor reduced T cell proliferation and activation both *in vivo* and *in vitro*, and reduced GVHD severity (76). Use of cucurbitacin E (CuE), a selective Stat3 inhibitor, reduced aGVHD severity in mice by reducing production of pro-inflammatory cytokines and Th17 cells (75).

These results were supported by cGVHD studies in mice, which showed that Stat3-deficient donor CD4^+^ T cells caused less GVHD than normal cells. This was due to reduced expansion and migration of donor T cells, not a change in the alloresponse of the T cells. Treg development was also enhanced by loss of Stat3 (78). Another study showed that loss of Stat3 in T cells enhanced natural Treg (nTreg) stability and promoted development of induced Tregs (iTregs) from CD4^+^ naive T cells. Again, Stat3-deficient CD4^+^ T cells protected against GVHD, although loss of Stat3 only in nTregs was not enough to stop GVHD (79). Suppression of Stat3 by injection of PIAS3 (protein inhibitor of activated Stat3) into mice to achieve PIAS3 overexpression resulted in reduced GVHD severity. This inhibitor also reduced the Th1 and Th17 subsets of T cells, while upregulating Th2 and Treg responses. Stat5 expression was also increased, which is discussed further below (80).

Grim19, a regulator of Stat3 expression, was found to decrease the severity of GVHD when overexpressed, largely due to suppression of Stat3 signaling and Th17 cell development, and enhanced Tregs and Stat5 expression (81). Mice treated with IL-22 were found to have more severe GVHD, again primarily due to increased phosphorylation of Stat3 and changes to T cell subsets such as Th1 and Th17 (82). Treatment of mice with KD05 (an inhibitor of Rock) resulted in amelioration of cGVHD symptoms, due in part to reduced Stat3 and increased Stat5 phosphorylation. Mice transplanted with T cells lacking Stat3 showed the same benefits, with lung function (lungs are a GVHD target) at the same level as healthy controls despite transplantation (83). Finally, use of nifuroxazide, a Stat3 inhibitor, resulted in reduced aGVHD severity and delayed death from symptoms. This inhibitor blocks activation of Stat3, and this led to reduced production of cytokines and increased Tregs (84). From this large body of data, it is clear that Stat3 impacts Treg and other helper T cell fates, cytokine production, and proliferation of alloreactive T cells.

Loss of Stat4 was found to induce lethal aGVHD, as was Stat6 loss in T cells. GVHD was rapidly lethal when Stat6 was lost, with almost all mice dead by day 7 post-transplant. Loss of Stat4 is typically associated with a defect in Th1 responses, while Stat6 loss is associated with defective Th2 responses (85). Overexpression of constitutively active Stat5b increased Tregs, producing a significant reduction in GVHD. These expanded Tregs were also hypersuppressive compared to those in WT mice. In addition, Stat5-overexpressing CD4^+^ T cells also caused less GVHD, because these cells produced less proinflammatory and more anti-inflammatory cytokines. These transgenic mice still maintained GVT effects, suggesting that enhancement of Stat5 signaling may be an effective therapy for GVHD (86).

These results were confirmed in a study of human T cells, which found that use of CAS 285986-31-4 (a STAT5 inhibitor) reduced iTregs (68). Finally, Stat6 was found to affect GVHD in mice. Treatment of T cells with IL-18 prior to allo-HSCT helped to reduce GVHD severity. When Stat6-deficient donor cells were used, this effect was removed, showing that Stat6 was required for this reduction in GVHD. IL-18 did not impact GVT effects, suggesting that Stat6 is important for GVHD but not GVT effects (87).

### 4.4 Aryl hydrocarbon receptor (Ahr)

Ahr is a transcription factor which binds to environmental contaminants that are aromatic hydrocarbons, such as dioxin or pollutants found in the air. Exposure to these ligands causes activation of Ahr, and transcription of downstream genes. Although Ahr has roles in many processes, it has recently been associated with changes to immune responses, such as immunosuppression caused by exposure to dioxin. Ligands found in food can also lead to immune changes through Ahr activation. Ahr is upregulated when T cells are activated, indicating a role in T cell functioning for this factor (88). Recent studies have identified a role for Ahr in GVHD, with mice receiving Ahr-/- T cells showing less severe aGVHD symptoms and better survival than mice receiving WT cells. This was primarily due to reduced proliferation of CD4^+^ T cells, increased peripheral Treg (pTreg) numbers in the colon, and less migration of CD8^+^ T cells to the spleen after transplant (89).

Importantly, loss of Ahr only impacted accumulation of T cells in the lymphoid tissues early on, but not in the target organs later on. No changes were seen in apoptosis levels, but proinflammatory cytokine production (IL-6, IFN-γ, TNF-α, and IL-1α) was reduced in the colon of the Ahr-/- T cell-receiving mice. GVHD attenuation was not due to loss of naive cells or a baseline increase in Tregs. In an experiment where mice received WT T cells or Ahr-/- T cells and tumor cells, the KO cells still cleared the tumor cells. However, twice as many KO cells were used as WT, because WT T cells caused severe GVHD, so the GVT effect may in fact be weakened in Ahr-/- T cells (89).

Interestingly, another group found seemingly contradictory results by feeding transplanted mice a diet low in diet-derived Ahr ligands. These mice had a reduction in ILC3 cells, leading to increased aGVHD severity and mortality (40). This work suggests that Ahr has a protective role in GVHD responses, whereas the results described above suggest that Ahr activation is detrimental. Earlier work by a different group showed that an engineered Ahr ligand called 10-Cl-BBQ induced a Treg phenotype in CD4^+^ T cells during a GVHD response. Foxp3 was not expressed by these cells, but the effect did require Ahr. Treatment of mice with this compound also inhibited development of GVHD (90). This work suggests that engagement of Ahr by at least some ligands has a protective effect during GVHD by inducing Treg development.

Finally, expression of Ido – an endogenous AhR ligand – was shown to be increased after allo-HSCT, and appears to help prevent/reduce lung pathology. Ido KO recipients had more lung damage, higher mortality, and more severe GVHD symptoms (91). Therefore, the role of Ahr is still unclear in GVHD, because some research shows a protective effect, while other work suggests a detrimental contribution. However, it is clear that Ahr receptor engagement affects peripheral Tregs, and it also impacts cell proliferation, migration, and proinflammatory cytokine production.

### 4.5 Nuclear factor (erythroid-derived 2)-like 2 (Nrf2)

Nrf2 is a transcription factor which controls the cellular redox and cellular stress pathways, and it is expressed ubiquitously. Nrf2 may contribute to survival of allotransplanted tissues (92). Recently, reports have shown that Nrf2 signaling plays a role in GVHD severity, but the results were seemingly contradictory (93–95). Loss of Nrf2 by genetic ablation resulted in reduced aGVHD when these T cells were allotransplanted, due to the presence of regulatory T cells expressing Helios, which were better able to persist when Nrf2 was ablated. Loss of Nrf2 did not affect proliferation, apoptosis, or activation of the T cells. In addition, CD8^+^ alloreactive T cells had impaired migration to the gut due to decreased expression of Lpam-1 (a homing receptor). Nrf2-deficient CD8^+^ T cells still had effective cytotoxic responses, and the GVT effect was preserved in mice receiving these cells (94).

However, another group allotransplanted mice and treated them with sulforaphane to induce Nrf2 activation. These mice had better long-term survival, less pathological damage, and reduced migration of T cells to peripheral tissues than mice receiving untreated T cells (95). Nrf2 activators such as arsenic trioxide (96) or dimethyl fumarate (93) reduced aGVHD severity but preserved GVL effects. These data suggest that activation of Nrf2 in the recipient reduces GVHD severity, while Nrf2 activation in donor T cells promotes GVHD damage. Therefore, it is unclear if Nrf2 expression is helpful or harmful during GVHD, but it is clear that Tregs and migration to peripheral tissues are affected by NRF2 changes.

## 5 Transcription factors which separate GVHD and GVL effects

### 5.1 Notch

Notch is a transcription factor which functions as a switch in T cell development. It controls fate decisions such as T cell versus B cell, CD4^+^ versus CD8^+^, and αβ versus γδ T cell fates (97). Notch acts as both a receptor and a transcription factor, because it is found in the cell membrane until binding of a ligand, at which point the intracellular portion enters the nucleus to affect transcription (98). There are several Notch receptors and several types of ligands, including Delta-group and Serrate-group molecules (97). One study showed that GVHD severity was reduced, and survival was improved when Notch signaling was blocked in donor T cells. This was due to reduced production of cytokines and effector molecules, but there was a slight increase in proliferation of the T cells. Migration to target organs was also reduced by Notch inhibition. GVT effects were still maintained, despite the changes to cytokine production (99).

A later study showed that GVHD from both CD4^+^ and CD8^+^ T cells was prevented by Notch inhibition. T cells lacking Notch showed a drop in IFN-γ production, decreased NF-κB signaling, increased Eomes expression, normal expansion, and normal T-bet expression. Essentially, these T cells were hyporesponsive but kept their potential for cytotoxicity (100). Blocking of all Notch signaling was found to be toxic, but Notch1/2 and Delta-like 1/4 (Dll1/4) were found to be most important for GVHD. Inhibition of Notch 1 still produced toxicity, but blocking of Dll1/4 successfully prevented GVHD without off-target effects. Antitumor functions were still preserved, just like with Notch inhibition in T cells, and temporary treatment was enough to provide stable protection (101–103). Targeting of Notch1/2 and Dll1/4 was also effective in cGVHD (104).

One group found that Notch1, Dll4, and Jagged-1 in donor T cells were reduced when Tregs were present. The authors suggest that the Tregs use a CD39-dependent mechanism to directly downregulate this signaling pathway in donor T cells, leading to improved survival and reduced GVHD severity (105). Another group suggested that Notch inhibition reduced GVHD by maintaining Treg function while lowering CD8^+^ T cell and APC activation and proliferation (106). More recent work by this group suggested that Tregs and central memory cells were increased when Notch was blocked, along with increased IFN-γ and lower TNF-α. They suggest that GVT effects were preserved because Fas/FasL components were not affected, and this pathway is primarily used for GVT(107). Finally, use of selective Notch inhibition in CD4^+^ T cells but not Tregs showed that the lack of Notch in CD4+ T cells was critical to stop GVHD. When Notch was lost only in Tregs, this effect was lost (108). Therefore, Notch affects T cell cytokine production, Treg expansion, and responsiveness to alloantigen, with loss resulting in reduced GVHD but maintained GVT effects (109, 110).

### 5.2 Promyelocytic leukemia zinc finger (Plzf)

Plzf has also been studied in T cells in the context of GVHD. This factor is critical for development of invariant natural killer T cells (iNKT) but is not expressed in mature CD4^+^ or CD8^+^ T cells. To study this factor, one group induced expression of Plzf using the Lck promoter to produce Plzf expression in mature CD4^+^/CD8^+^ T cells (called “Plzf-TG”). They found that these knock-in cells have a memory and innate-like phenotype without proliferation or stimulation by antigen. In addition, these mice had higher frequencies of Tregs, similar homing potential (by Ccr7 expression), higher IFN-γ and TNF-α levels, and less IL-2 production at baseline. Mice transplanted with these Plzf-TG cells had reduced GVHD severity as well as reduced pathology in small intestine, large intestine, and skin (but not liver). This attenuation of GVHD was primarily driven by CD4^+^ T cells, which typically cause more severe disease (111).

Plzf-TG T cells underwent apoptosis more frequently even though they were still capable of being alloactivated like WT cells. Once these cells were alloactivated, they produced the same amounts of IFN-γ and TNF-α as WT cells, but more IL-4 and less IL-2 than WT. Th2 cells were more prominently produced from Plzf-TG mice, due to enhanced IL-4 production and Gata-3 expression. Finally, expression of Plzf in these T cells resulted in better tumor clearance and survival. Therefore, Plzf expression contributed to regulation of apoptosis, Th2 cell fate, and Tregs, leading to reduced GVHD but maintained GVT effects (111). However, because the expression of Plzf was induced in these cells but is not naturally present, it is unknown how these results may extrapolate to humans.

### 5.3 Nuclear factor kappa-light-chain-enhancer of activated B cells (Nf-κb)

Nf-κb is a transcription factor complex which controls DNA transcription, cell survival, cytokine production, and responses to a variety of stimuli. Nf-κb can refer to the complex of factors or to the members Nf-κb1 or Nf-κb2. Early reports suggested a role for Nf-κb in GVHD by examining the use of Nf-κb inhibitors. One group tested the inhibitors bortezomib and PS-1145 and found that both could reduce GVHD severity in mice. This reduction occurred without disruption of engraftment and was primarily due to reduced cytokine production in recipients. Interestingly, use of bortezomib (which is not Nf-κb specific) for longer time periods caused more severe damage, while use of PS-1145 (NF-κB specific) gave even better protection from GVHD than a shorter course (112). These results with PS-1145 were confirmed by another group, which found that the inhibitor allowed for reduced GVHD and responses to alloantigen, but maintained responses to mitogens (113). Another study found that use of the cell cycle inhibitor (R)-roscovitine reduced GVHD severity, mainly due to reduced T cell expansion and TNF-α-driven Nf-κb activation (114).

Activation of Nf-κb by TNF-α relies on Nf-κb-inducing kinase (Nik). Lack of Nik in T cells that were allotransplanted into recipient mice prevented GVHD from developing. Overactivation of Nik in T cells led to fatal autoimmune responses due to excessive function of effector T cells and lack of suppressive function among Tregs. Therefore, changes in Nik can alter Nf-κb activation levels to disrupt alloreactive T cell effector function and Treg development (115). Rosiglitazone (a Pparγ agonist) was also examined in this context. This drug was found to inhibit GVHD, mainly by reducing cytokine production, increasing Tregs, and decreasing CD8^+^ T cells (from both donor and host). Rosiglitazone also prevented Nf-κb activation typically seen in GVHD, leading to a protective effect (116).

Dehydroxymethylepoxyquinomicin (DHMEQ) was created as an Nf-κb inhibitor, and was also found to suppress GVHD in mice(117). Tranilast, an Nf-κb and Txnip inhibitor, was found to reduce cGVHD development in MHC-matched, miHA-mismatched mice (118). A very recent study of SNPs in the NF-κB1 gene found that two specific SNPs in this gene were associated with aGVHD and mortality after transplant (119). Therefore, Nf-κb clearly plays a role in GVHD, with primary effects on T cell proliferation, cytokine production, and Treg differentiation.

The Nf-κb family is composed of five major subunits, one of which is c-Rel (120). This factor plays a role in T cell alloresponses, as well as in canonical immunity. Several reports showed that loss of c-Rel in T cells leads to attenuated GVHD with preserved GVT responses (121–123). In a study using T cells lacking c-Rel, the reduction in GVHD was primarily due to reduced expansion of T cells in secondary lymphoid organs, as well as a reduced ability to migrate to target organs (due to lower chemokine receptor expression). GVHD was reduced in both major and minor mismatch models. These cells had reduced Th1 and Th17 fate differentiation and increased Treg fate differentiation during alloactivation. Th1 cytokine production was reduced, as was production of TNF-α. In addition, these c-Rel KO cells had higher rates of apoptosis than WT cells. However, c-Rel KO T cells were still able to perform GVT responses, although this ability was partly reduced (122).

Support for these findings came from use of a small molecule inhibitor of c-Rel. In this study, mice receiving WT T cells showed increased expression of c-Rel post-transplant. This group identified pyrimidinetrione and its derivatives as inhibitors of c-Rel which were highly specific and highly potent. Treatment of T cells prior to transplant with these c-Rel inhibitors reduced T cell expansion in the recipient mouse and reduced GVHD, even though c-Rel levels returned to normal within 4 days. These inhibitors did not prevent GVT effects from occurring (123). These studies show that c-Rel plays a critical role in GVHD processes by controlling T cell migration, expansion, cytokine production, and fate decisions.

Relb is another subunit of Nf-κb which plays a role in GVHD; however, this factor is primarily important for APCs during this response. Relb within APCs (specifically dendritic cells, DCs) was found to be critical for GVHD occurrence and persistence, as well as Th1 cell expansion. Loss of Relb did not affect Treg expansion or functioning (124). (Therefore, while it is not highly expressed in T cells during the alloresponse, RelB in APCs does play a role in how T cells can respond in this context.

Finally, the Rela subunit of Nf-κb has been shown to play a role in GVHD through its binding. Use of I-BET151 and JQ1, two inhibitors of bromodomain binding and histone acetylation, led to reduced GVHD in mice. These mice had maintained GVT effects, despite reduced cytokine production and proliferation of T cells. The authors suggested that these effects were mediated by disrupted binding of Rela with BRD4 (125). Thus, other components of the Nf-κb family are also important for GVHD.

### 5.4 Janus kinase (Jaks)

Jak family transcription factors are involved in the Jak/Stat signaling pathway, which controls T cell cytokine and growth factor signaling, inflammatory responses, and innate and adaptive responses (126). A large body of evidence has emerged regarding the JAK1/2 inhibitor ruxolitinib, which has been shown in research and clinical trials to reduce GVHD severity (127–129). This reduction in disease was due to reduced proinflammatory cytokine production (130), as well as reduced chemokine receptor expression, which impacts T cell migration (131). Ruxolitinib also decreased T cell proliferation and Treg differentiation in some reports (132, 133). GVT effects were preserved in mice treated with ruxolitinib (131), Tofacitinib, another Jak inhibitor, was found to prevent GVHD and also to reverse existing disease in mice. This was primarily due to impaired proliferation and activation of alloreactive CD8^+^ T cells in the mouse. This effect was also seen on human CD8^+^ T cells (134). An ocular inhibitor of JAK and SYK called R348 was tested in a pilot study, but was unsuccessful at inducing beneficial results for ocular GVHD (135).

One group used genetic ablation of Jak2 in donor T cells to examine the role of this specific Jak in GVHD. Loss of Jak2 reduced GVHD severity but preserved GVT effects. This was mainly due to reduced Th1 differentiation, and increased Th2 and Treg responses. The same group used pacritinib to target Jak2 and found that this drug reduced GVHD and rejection of xenogeneic skin grafts in mice. Again, GVT effects were preserved in treated mice. The same changes in cell fate were observed as with Jak ablation (136). Recently, a new clinical trial was performed to evaluate itacitinib, a JAK1 selective inhibitor, in aGVHD patients. This drug was successful in preclinical models, and it showed benefit for 75 percent of patients in the study (response rate was 70.6 percent among those whose GVHD was refractory to steroids). In addition, all of the study patients receiving the drug were able to decrease usage of corticosteroids (137). These data suggest that targeting of Jak1 and/or Jak2 are successful strategies for limiting GVHD.

### 5.5 Enhancer of zeste homolog 2 (Ezh2)

Ezh2 is another T cell factor which has been shown to be important for GVHD. Initially, investigators suspected this factor would be critical because it methylates histone 3 at lysine 7, thereby controlling gene expression. However, inhibiting only this methylation process and not Ezh2 itself did not reduce GVHD (3, 138). Inhibiting Ezh2 directly (138), or by destabilizing the Ezh2 protein through Hsp90 inhibition (3) did reduce murine GVHD. This effect was reversed by manually adding Ezh2 protein back into the T cells (139) Another study using T cell conditional Ezh2 deletion (Ezh2 flox x CD4cre) showed reduced GVHD in mice with the deletion (140). The authors reported that initially these knockout cells were more activated, but later on they lost control of proliferation/expansion and could not properly differentiate into IFN-γ-producing effector cells (140).

Several reports also showed that while loss of Ezh2 reduced GVHD, it maintained GVT effects (3, 140). Ezh2 is also important for expression of T-bet and Stat4. Importantly, more recent reports using human T cells in mice for a xenogeneic GVHD model showed no reduction in GVHD when EZH2 was lost, raising the question of whether this factor is also important in human disease (138). However, at least in mice, Ezh2 appears to directly control continued proliferation and IFN-γ production capability of the cells, while preserving GVT functions.

### 5.6 Hypoxia-inducible factor-1 alpha (Hif-1α)

Hif-1α serves as a metabolic sensor in CD4^+^ T cells (141), and enhances CD8^+^ T cell cytotoxic functions (142). Hif-1α also reduces development of Treg cells when activated (141). In chronic GVHD patients, bronchiolitis obliterans (a complication involving the lungs) was associated with higher expression of HIF-1α (143), suggesting a role for the factor in GVHD. Echinomycin is a small-molecule HIF-1α inhibitor which reduces binding of targets to the transcription factor. Use of this inhibitor decreased Th17 cell development and survival, in a manner similar to that seen when HIF-1α was ablated in CD4^+^ T cells (144).

Echinomycin treatment also reduced GVHD in mice receiving allo-HSCT. This was due to an increase in Tregs, as well as reduced Th1 and Th17 cells. However, GVL effects were still preserved, and echinomycin did not inhibit tumor cells directly at normal levels (141). These results were recently confirmed in a humanized mouse model of aGVHD (142). Thus, Hif-1α inhibition may have therapeutic value in place of Treg infusions by enhancing donor Treg expansion post allo-HSCT.

### 5.7 Nuclear factor of activated T cells (Nfat)

Nfat represents a family of four transcription factor members, all of which are regulated by calcium and calcineurin. The role of Nfat factors varies depending on the cell type, and even the cell subset (i.e. Tregs versus activated T cells). Interestingly, drugs such as cyclosporine or tacrolimus which are used to treat GVHD suppress GVT effects, as well as inhibit calcineurin (thereby suppressing NFAT). One group showed that loss of Nfat in T cells resulted in reduced GVHD severity with maintained GVT effects. Ablation of Nfat1, Nfat2, or Nfat1/2 were examined with the same results (145).

This reduction in GVHD resulted from reduced T cell proliferation and migration, as well as reduced production of effector cytokines. Loss of Nfat also increased the frequency of Tregs derived from donor cells, which were still suppressive. Despite the changes in T cell function, GVT effects and OVA-specific immune recall responses (i.e. T cell memory to antigen) were preserved in Nfat KO cells (145). These results were supported by later work showing that downregulation of Nfat by overexpression of Grim19 reduced the severity of GVHD (81).

Additionally, the Runt-related transcriptional factor (Runx) family of factors has been shown to directly transactivate Nfatc2. One group showed that a novel Runx inhibitor, Chb-M’, nearly eliminated all signs of GVHD in a xenogeneic mouse model. This alleviation was due to a decrease in CD4+ T cell proliferation and GVHD-associated cytokines. Chb-M’ also suppressed Nfatc2 expression, suggesting that the Runx/Nfatc2 axis may be a target for GVHD amelioration (146). Therefore, loss of Nfat reduced GVHD while maintaining GVT, largely through effects on migration, cytokine production, proliferation, and Treg differentiation.

### 5.8 Friend virus leukemia integration 1 (Fli-1)

Friend virus leukemia integration 1 (Fli-1) plays a role in T cell proliferation, inflammatory cytokines and chemokine receptor production, and CD8^+^ T cell responses to infection and tumors. One group examined the role of Fli-1 in GVHD and GVT using T cell-specific deletion of Fli-1 and pharmacologic inhibitors of Fli-1. Interestingly, heterozygous loss of Fli-1 in T cells led to the mildest cGVHD and aGVHD symptoms (compared to WT or homozygous deletion). Differentiation of CD4^+^ T cells to Treg and Tfh states was also affected. When inhibitors were used, loss of Fli-1 alleviated GVHD while maintaining the GVT effect (147). Therefore, Fli-1 affects alloreactive CD4^+^ T cell differentiation and activation.

### 5.10 Basic helix-loop-helix family member e40 (Bhlhe40)

Basic helix-loop-helix family member e40 (Bhlhe40) has been shown to regulate GM-CSF production and support pathogenicity in neuroinflammation (in murine models). Increased expression of this factor was identified in a CD4^+^ CD11c^+^ T cell population found to be important for early inflammation events in GVHD in the gastrointestinal tract. Mice allotransplanted with Bhlhe40^-/-^ CD4^+^ T cells showed significantly improved weight recovery and survival, as well as reduced pathological damage in the colon, compared with mice receiving WT cells. Follow-up experiments with GM-CSF-deficient T cells supported the idea that loss of Bhlhe40 affects GVHD through control of GM-CSF production (148). Therefore, Bhlhe40 plays a role in alloreactive CD4^+^ T cell cytokine production, thereby affecting GVHD symptoms,

## 6 Regulatory networks in GVHD and GVL

As shown above, a wide variety of transcription factors have been studied for their effects on GVHD and GVL processes. However, understanding of the regulatory networks which govern GVHD, GVL, and the separation of these two processes is seriously lacking.

### 6.1 T cell factor 1 (Tcf-1)

Tcf-1 is a T-cell specific transcription factor that is critical for T cell development. The double-negative stages of development in the thymus require expression of Tcf-1, allowing the thymocyte to proceed correctly (149–154). Although Tcf-1 has been previously studied in canonical activation as well (154–157), the role of Tcf-1 in GVHD or GVT effects has not been extensively studied.

Expression of the factors Eomes and T-bet was increased in CD8^+^ T cells from Tcf-1 cKO mice. This suggests that Tcf-1 normally suppresses Eomes and T-bet in CD8^+^ T cells, thereby contributing to regulation of alloactivation through these factors. CD8^+^ and CD4^+^ T cells lacking Tcf-1 also showed higher activation (by CD44 and CD122) and changes to memory phenotypes. We found that loss of TCF-1 specifically in T cells (CD4^+^ and CD8^+^) lead to reduced GVHD severity and persistence. Allotransplanted mice receiving Tcf-1-deficient T cells had lower GVHD scores, less weight loss, better survival, and were able to resolve GVHD (instead of developing persistent disease) (52, 158).

For CD4^+^ donor T cells, the reduced GVHD severity was likely due to reduced cytokine production and survival, and changes to gene expression and chemokine/chemokine receptor expression (158). For donor CD8^+^ T cells, the reduction in disease was likely primarily due to reduced cytokine production, increased exhaustion, increased central memory cells pre-transplant, and enhanced utilization of Nkg2d to mediate cytotoxicity (52). Gene expression and expression of chemokines/chemokine receptors were altered in donor cells by loss of Tcf-1 (52, 158).

Despite reductions in GVHD severity, mice receiving Tcf-1 cKO T cells still mediated GVT effects, and tumor burden was maintained at the same level as in mice receiving WT T cells, indicating no defect in GVT effects. This is likely due to increases in perforin, granzyme b, and Nkg2d utilization in Tcf-1 cKO donor CD8^+^ T cells (52). Therefore, Tcf-1 affects T cell phenotype, exhaustion, proliferation, survival, gene expression, chemokine/chemokine receptor expression, and cytokine/cytotoxic mediator production to affect GVHD and GVT (52, 158).

## 7 Discussion

**Figure 2:**
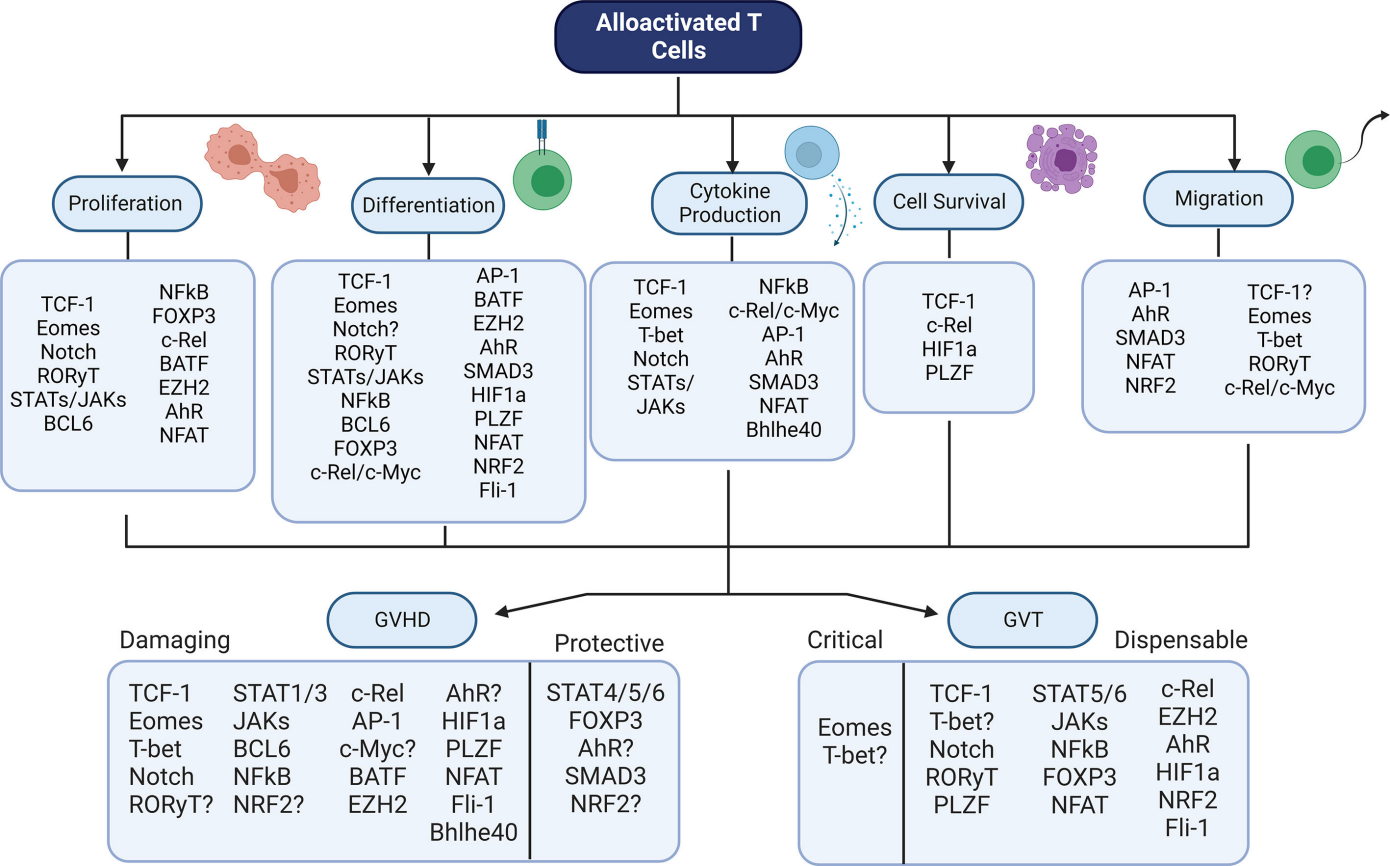
Summary Model - Transcriptional Control of GVHD and GVT. Numerous transcription factors have an impact on T cell function during alloactivation. Alterations in these factors leads to changes in GVHD and/or GVT outcomes. Shown here is the contribution of each factor discussed to each of the major T cell functions, as well as the factor’s role in GVHD (damaging or protective) and GVT (dispensable or critical). Figure created using BioRender.com.

There is a wide array of transcription factors that contribute to GVHD and GVT alloresponses after allo-HSCT (**Table 1**). Although many of these factors have been studied through genetic or pharmaceutical abrogation, their role in these processes is still not fully certain. In addition, it is interesting to note that the role of these factors in controlling other factors – the basis of a transcriptional regulatory network – has not yet been discussed in many cases. In order to fully understand how best to target T cell factors to separate GVHD from GVT, it is essential to understand the downstream effects of abrogation of a single factor. Therefore, the model pictured below (**Figure 2**) may give an understanding of how each factor relates to GVHD/GVT, but it is still incomplete. Information about how each factor relates to each other factor must still be added as research progresses in this area.

**Table 1:** Summary of transcription factor involvement in GVHD/GVT.

Factor	Function Impacted	Role	Impact of Loss
Tcf-1	Cytokine Production, Survival, Proliferation, Chemokines, Gene Expression	Promotes T cell proliferation/survival, controls cytokines/mediators, affects expression of chemokines/receptors, regulates Eomes/T-bet (CD8+ only)	Reduced GVHD, maintained GVT
Eomes	Proliferation, Migration, Cytokine Production, Differentiation	Controls T cell migration and proliferation, affects production of cytokines/cytotoxic mediators, impacts Treg fate	Reduced GVHD, reduced GVT
T-bet	Migration, Cytokine Production	Controls T cell migration and production of Th1 cytokines	Reduced GVHD, reduced/maintained GVT
Notch	Cytokine Production, Proliferation, Differentiation	Controls T cell fate, cytokine production, response to alloantigen, and Treg expansion	Reduced GVHD, maintained GVT
Rorγt	Migration, Proliferation, Differentiation	Drives Th17 cell fate, proliferation, and migration	No change/reduced GVHD, maintained GVT
Stats	Differentiation, Proliferation, Cytokine Production	Controls Treg/Th subset fate, cytokine production, and proliferation	Reduced (STAT1/3) or enhanced (STAT4/5/6) GVHD, maintained GVT (STAT5/6)
Jaks	Differentiation, Proliferation, Cytokine Production	Controls Th1/Th2 polarization, Treg cell fate, proinflammatory cytokines, and T cell proliferation	Reduced GVHD, maintained GVT
Bcl6	Differentiation, Proliferation	Regulates GC formation by Tfh cells and effector CD4+ expansion	Reduced cGVHD
Nf-κb	Differentiation, Cytokine Production, Proliferation	Regulates Treg development, cytokines, and T cell expansion	Reduced GVHD, maintained GVT
Foxp3 (and Tregs)	Differentiation, Proliferation	Lineage factor for Tregs, stability regulates Treg function	Increased GVHD, maintained GVT
c-Rel	Differentiation, Proliferation, Apoptosis, Migration, Cytokine Production	Regulates migration to target organs, formation of Tregs/Th1/Th17, cytokine production, and apoptosis	Reduced GVHD, maintained GVT
c-Fos/c-Jun (Ap-1) and c-Myc	Differentiation, Migration, Cytokine Production	May regulate migration, Treg fate, and cytokine production	Reduced GVHD (human studies show possible contradiction)
Batf	Differentiation, Proliferation	Controls IL-7/GM-CSF-producing T cells	Reduced GVHD
Ezh2	Proliferation, Differentiation	Regulates long-term proliferation/expansion, promotes differentiation to IFN-γ+ effector fate	Reduced GVHD, maintained GVT
Ahr	Proliferation, Migration, Cytokine Production, Differentiation	Regulates pTregs, CD4+ proliferation, CD8+ migration, and proinflammatory cytokine production	Reduced/increased GVHD, maintained GVT
Smad3	Migration, Differentiation, Cytokine Production	Controls Th fate decisions, T cell migration, and proinflammatory cytokines	Reduced allograft rejection, enhanced GVHD
Hif1α	Differentiation, Survival	Inhibits Tregs and enhances Th17/Th1 cells	Reduced GVHD, maintained GVT
Plzf	Apoptosis, Differentiation	Regulates apoptosis, Th2 fate, and Treg cells	Reduced GVHD, maintained GVT
Nfat	Proliferation, Migration, Cytokine Production, Differentiation	Regulates migration, Treg development, cytokine production, and proliferation of T cells	Reduced GVHD, maintained GVT
Nrf2	Differentiation, Migration	Inhibits Helios+ Tregs, controls migration to periphery	Reduced/enhanced GVHD ()?, maintained GVT
Fli-1	Differentiation, Activation	Regulates CD4+ T cell differentiation to Treg/Tfh fates, regulates effector CD4+ T cells	Reduced GVHD, maintained GVT
Bhlhe40	Cytokine Production	Regulates GM-CSF production in CD4+ T cells	Reduced GVHD

Based on the major functions of alloactivated T cells (**Figure 1**), there are a variety of ways in which GVHD and GVL can be separated theoretically. First, the time frame of cell survival following allotransplantation can potentially separate GVHD and GVL. GVL occurs rapidly following transplant, as tumor cells are destroyed by the grafted cells. However, GVHD is an ongoing process which requires continued activity of the alloreactive cells to be sustained (159–161). Therefore, if the allotransplanted cells have limited survival, then GVHD may be reduced or alleviated while GVL effect persist. Similarly, if the allotransplanted cells survive but have limited function - through exhaustion, cell suicide genetic programming, expression of suppressive markers - then GVHD may be alleviated (or not persist) while GVL effects are maintained.

Cell migration is another essential process for GVHD, because alloreactive cells must migrate to the tissues (especially liver, lungs, small intestine, and skin) in order to cause damage (162). If the cells cannot migrate properly to the target tissues, then GVHD will be alleviated. GVL effects against blood cancers such as leukemias do not require migration to tissues, because the cancer cells circulate in the blood and encounter the alloreactive cells in circulation. Therefore, altered migration can separate GVHD and GVL effects.

Changes in differentiation (including production of cytokines/cytotoxic mediators and gene expression) can also potentially separate GVHD from GVL effects. CD44hi T cells have been reported to abrogate or reduce the severity of GVHD (2, 163). CD4 T cells are also known to cause more severe GVHD than CD8 T cells (164, 165). Production of pro-inflammatory cytokines also exacerbates GVHD (7, 166).

Transcription factors present a unique opportunity for modulation of GVHD and GVL, precisely because they function as part of regulatory networks. Modulation of one transcription factor can have downstream, upstream, and horizontal effects on various pathways. This also allows for a greater variety of potential targets, because if a particular factor is not druggable, others in the same pathway or network may be a possible target instead, with the same effects. As research in allotransplantation continues, the focus should be on identifying which factors are involved in regulatory networks that control alloactivated T cell functions. Future work should emphasize understanding of these networks, as this is a critical unmet need despite the fact that the transcription factors involved in these processes do not function independently of such networks; rather, the effects of modulation of one factor cascade downstream and outwards. Improved understanding of how transcription factor modulation affects the network as a whole will enable more precise control of GVHD and GVL processes, thereby improving research into allotransplantation outcomes.

## Author contributions

RH and MK developed the concept for this review. RH performed the literature search and wrote the manuscript. MK provided critical review and scientific guidance. All authors contributed to the article and approved the submitted version.
